# Effect of the rs2259816 polymorphism in the HNF1A gene on circulating levels of c-reactive protein and coronary artery disease (the ludwigshafen risk and cardiovascular health study)

**DOI:** 10.1186/1471-2350-11-157

**Published:** 2010-11-09

**Authors:** Marcus E Kleber, Tanja B Grammer, Wilfried Renner, Winfried März

**Affiliations:** 1 LURIC Study nonprofit LLC, Freiburg, Germany; 2 Synlab Centre of Laboratory Diagnostics, Heidelberg, Germany; 3 Clinical Institute of Medical and Chemical Laboratory Diagnostics, Medical University of Graz, Graz, Austria; 4 Institute of Public Health, Social and Preventive Medicine, Medical Faculty of Mannheim, University of Heidelberg, Mannheim, Germany

## Abstract

**Background:**

C-reactive protein is a well established marker of inflammation and has been used to predict future cardiovascular disease. It is still controversial if it plays an active role in the development of cardiovascular disease. Recently, polymorphisms in the gene for HNF1α have been linked to the levels of C-reactive protein and coronary artery disease.

**Methods:**

We investigated the association of the rs2259816 polymorphism in the HNF1A gene with the circulating level of C-reactive protein and the hazard of coronary artery disease in the LURIC Study cohort.

**Results:**

Compared to CC homozygotes, the level of C-reactive protein was decreased in carriers of at least one A-allele. Each A-allele decreased CRP by approximately 15%. The odds ratio for coronary artery disease was only very slightly increased in carriers of the A-allele and this association did not reach statistical significance.

**Conclusions:**

In the LURIC Study cohort the A-allele of rs2259816 is associated with decreased CRP but not with coronary artery disease.

## Background

C-reactive protein (CRP) is a well established biochemical marker of inflammation and has been used to predict future cardiovascular disease [1-3]. As its level is increased in patients suffering from coronary artery disease (CAD) the idea has been put forward that it might play an active role in the development of the disease. Although numerous studies have been conducted, this issue has not been finally settled yet [4-6]. Most of these studies didn't find an association between CRP and CAD [7-13] whereas a few did present some evidence in favor of this idea [14-16].

A recent meta-analysis has shown that associations of CRP with ischemic vascular disease depend considerably on conventional risk factors and other markers of inflammation making a causal role of CRP in the development of CAD also unlikely [17].

Genetic factors have been estimated to have a great influence on the variance in plasma CRP level [18-20]. A number of polymorphisms of the CRP gene (MIM 123260) or its promoter that act in this way have been described so far [8-11,14-16,21-32] but they only account for a minor part of the assumed heritability.

Recently, polymorphisms in the HNF1A gene (also known asTCF1, MIM 142410) have been linked to the levels of C-reactive protein and coronary artery disease [33-36]. This gene encodes the transcription factor hepatocyte nuclear factor (HNF)-1α which regulates the transcription of numerous genes in miscellaneous tissues, including genes that are expressed exclusively in the liver [37-40]. The CRP gene promoter contains a HNF-1α binding site which is involved in the regulation of basal and constitutive CRP synthesis in the liver [41].

In our study we attempted to confirm the reported association of rs2259816 to CRP and CAD in the LURIC Study cohort.

## Methods

### Study design and participants

The **Lu**dwigshafen **Ri**sk and **C**ardiovascular Health (LURIC) study includes consecutive white patients hospitalized for coronary angiography between June 1997 and May 2001. A detailed description of LURIC has been published [42]. The study was approved by the ethics review committee at the "Landesärztekammer Rheinland-Pfalz" (Mainz, Germany). Written informed consent was obtained from each of the participants. Clinical indications for angiography were chest pain or non-invasive tests consistent with myocardial ischemia. To limit clinical heterogeneity, individuals suffering from acute illness other than acute coronary syndromes, chronic non-cardiac diseases and a history of malignancy within the five past years were excluded.

CAD has been defined angiographically using the maximum luminal narrowing estimated by visual analysis. CAD was defined as the presence of a visible luminal narrowing (> 20% stenosis) in at least one of 15 coronary segments according to a classification of the American Heart Association [43]. Individuals with stenosis < 20% were considered as not having CAD. To examine the impact of other definitions of CAD on the current analysis, we provisionally used the presence of one stenosis > 50% (n = 2158) as a criterion. MI was defined as evidence for any MI (acute, previous, ST elevation MI, STEMI, or non ST elevation MI, NSTEMI). Acute MI was defined as a MI that had occurred within the four weeks prior to enrolment into LURIC. A previous MI was diagnosed if a MI had been survived for more than one month before enrolment into LURIC. A definite STEMI was diagnosed if typical ECG changes were present along with prolonged chest pain refractory to sublingual nitrates and/or enzyme or troponin T elevations. NSTEMI was diagnosed if symptoms and/or enzyme criteria, but not the ECG criteria for STEMI were met. Previous MI was also graded as definite if a hospitalisation with a discharge diagnosis of MI was documented.

Among the 3113 individuals in whom rs2259816 genotypes were available, 665 (21.4%) had no angiographic CAD while 2448 (78.6%) had CAD.

Diabetes mellitus was diagnosed according to the criteria of the American Diabetes Association [44]. Further, individuals with a history of diabetes or treatment with oral antidiabetics or insulin were considered diabetic. Hypertension was defined as a systolic and/or diastolic blood pressure ≥ 140 and/or ≥ 90 mm Hg or a significant history of hypertension.

### Laboratory Procedures

Fasting blood samples were obtained by venipuncture in the early morning. 'Sensitive' C-reactive protein was measured by immunonephelometry on a Behring Nephelometer II (N High Sensitivity CRP, Dade Behring, Marburg, Germany) after completion of the patient recruitment in 2001 in samples stored at -80°C. In the C-reactive protein assay used, the limit of detection for C-reactive protein is 0.17 mg/L; it is linear up to 500 mg/L. The lowest and the highest C-reactive protein concentrations encountered in this study were 0.17 and 269 mg/L, respectively. Blood glucose was determined enzymatically using the hexokinase/glucose-6-phosphate dehydrogenase method (Roche Diagnostics, Mannheim, Germany). Lipoproteins were separated by a combined ultracentrifugation-precipitation method (β-quantification) [42]. Cholesterol and triglycerides were measured with enzymatic reagents from WAKO (Neuss, Germany) on a WAKO R30 or Olympus AU640 analyser [42].

### Analysis of the rs2259816 genotype

Genomic DNA was prepared from EDTA anticoagulated peripheral blood by using a common salting-out procedure. The C/A polymorphism rs2259816 was genotyped by a 5' exonuclease assay (Taqman^®^). Primer and probe sets were designed and manufactured using Applied Biosystem's 'Assay-by-Design' custom service.

### Statistics

Continuous variables were first tested for normality and then compared between groups by univariate analysis of variance (ANOVA). CRP was not normally distributed and had to be logarithmically transformed before performing ANOVA. Co-variables were used as indicated.

In models assuming a co-dominant (additive) effect of the alleles, genotypes were coded as 0, 1, and 2, respectively, and genotypes were either treated as interval-scaled or categorical variables, the most frequent genotype being considered as the reference category in the latter case. When assuming a dominant effect, the genotype CC was coded as 0, and the combined remaining ones were coded as 1. When assuming a recessive effect, the genotypes CC and AC were coded as 0, genotype AA as 1. Further, the estimated marginal means of the dependent variables along with their 95% confident intervals (CI) are reported in the ANOVA procedures and the least significant difference t-test was used for post hoc comparisons. Estimated marginal means are not observed means; rather they represent predicted means estimated at the co-variables held at their respective actual means. Finally, we analyzed the association between rs2259816 genotypes and angiographic CAD in an analogous fashion by logistic regression. Multivariable adjustment was carried out in two steps, first for sex and age, and then, in addition, for cardiovascular risk factors (body mass index, diabetes mellitus, hypertension, smoking, LDL-C, HDL-C and TG. All statistical tests were two-sided. P < 0.05 was considered statistically significant. The SPSS 16.0 statistical package (SPSS Inc., Chicago, IL, USA) was used.

## Results

### Study participants

We included 3113 subjects in the current analysis. Compared to the control group without CAD, patients with angiographic CAD were significantly older; current or past smoking, diabetes mellitus, and hypertension were more prevalent. CAD patients had higher systolic blood pressure, higher fasting glucose, higher triglycerides and lower HDL-C. Crude LDL-C concentrations were higher in controls, due to the fact that 57.1% of CAD patients were treated with lipid-lowering drugs compared to 18.3% of controls. When we accounted for the use of lipid-lowering drugs, age, and gender, however, LDL-C was significantly higher in CAD patients (adjusted mean 118 mg/dl) than in controls (adjusted mean 113 mg/dl). Body mass index and diastolic blood pressure were similar between patients and controls (Table 1).

**Table 1:** Baseline characteristics and risk factors in patients with angiographically proven CAD as compared to controls without CAD.

	No CAD	CAD	*P*^1^
	(n = 665)	(n = 2448)	
Age (years) means ± SD	58 ± 12	64 ± 10	< 0.001
Body mass index (kg/m^2^) means ± SD	27 ± 4	28 ± 4	n.s.
Smoker (former and current)	324 (49%)	1675 (68%)	
Diabetes mellitus	125 (19%)	872 (36%)	
Systemic Hypertension	417 (63%)	1841 (75%)	

Systolic blood pressure (mm Hg) means ± SD	136 ± 22	142 ± 24	0.014^3^
Diastolic blood pressure (mm Hg) means ± SD	80 ± 11	81 ± 11	n.s.
Fasting blood glucose (mg/dl) means ± SD	105 ± 28	116 ± 37	< 0.001
LDL-C(mg/dl) means ± SD	120 ± 31	116 ± 35	0.001^4^
HDL-C (mg/dl) means ± SD	43 ± 12	38 ± 10	< 0.001^4^
Triglycerides (mg/dl)median (25^th ^and 75^th ^percentile)	132 (97-194)	150 (113-202)	< 0.001

^1Analysis of variance or logistic regression, respectively, adjusted for age and gender.2Analysis of variance adjusted for gender only.3Analysis of variance additionally adjusted for use of lipid-lowering drugs, beta blockers, ACE inhibitors, AT1 receptor antagonists, calcium channel blockers and diuretics.^

^4Analysis of variance additionally adjusted for use of lipid-lowering drugs.^

### Association of rs2259816 with CRP

**Figure 1:**
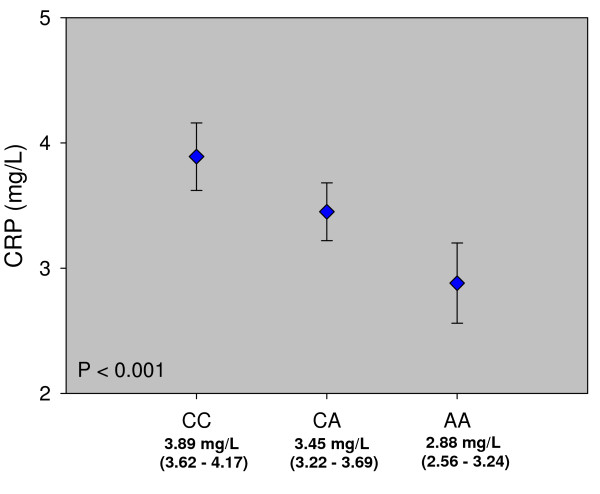
**Levels of CRP according to rs2259816 genotype**. CRP by rs2259816 genotype. Displayed are the estimated marginal means (± 95% confidence intervals) of a linear model (ANOVA) adjusted for age, gender, smoking (never, former, current), body mass index, diabetes mellitus, hypertension, LDL-C, HDL-C and TG. The A-allele of rs2259816 was associated with significantly lower CRP (*P <*0.001)

To analyze the association of levels of CRP with rs2259816 genotype, we used analysis of variance in which the genotype groups (defined by the presence or the absence of an A-allele) were included as main effects; statistical adjustments were made for age, sex and cardiovascular risk factors, namely body mass index, diabetes mellitus, hypertension, smoking, LDL-C, HDL-C and TG (Figure 1).

Compared to CC homozygotes, carriers of the A-allele had significantly lower plasma concentration of CRP (*P *< 0.001). The circulating plasma concentration decreased from 3.88 mg/l (3.63 - 4.16) in CC homozygotes to 3.43 mg/l (3.21 - 3.65) in heterozygotes and to 2.95 mg/l (2.64 - 3.31) in AA homozygotes. As statins also lower CRP we repeated our analysis including only patients not receiving lipid lowering therapy but this yielded basically the same results (data not shown).

### rs2259816 and coronary artery disease

Compared to CC homozygotes, the prevalence of CAD was basically the same in CA heterozygotes with OR ranging from 1.005 to 1.038, depending on the statistical model (Table 2). Even for AA homozygotes there was only a slightly higher increase with OR ranging from 1.050 to 1.083. Both associations were not statistically significant. There was no significant association between rs2259816 and CAD in any of the tested models either.

**Table 2:** Odds ratios (OR) for angiographic CAD according to the rs2259816 genotype.

rs2259816 genotype	Model 1 OR (95% CI)	*P*	Model 2 OR (95% CI)	*P*	Model 3 OR (95% CI)	*P*
CC (n = 1264)	1.0^reference^		1.0^reference^		1.0^reference^	
CA^1 ^(n = 1395)	1.005 (0.835 - 1.210)	0.957	1.013 (0.832 - 1.232)	0.900	1.038 (0.847 - 1.272)	0.717
AA^1 ^(n = 454)	1.050 (0.807 - 1.367)	0.716	1.045 (0.792 - 1.380)	0.755	1.083 (0.811 - 1.445)	0.590
CC vs CA vs AA	1.020 (0.901 - 1.154)	0.755	1.020 (0.895 - 1.162)	0.766	1.040 (0.908 - 1.191)	0.572
CC vs CA + AA	1.016 (0.835 - 1.209)	0.860	1.021 (0.848 - 1.227)	0.829	1.049 (0.866 - 1.270)	0.626
CC + CA vs AA	1.047 (0.820 - 1.338)	0.712	1.038 (0.802 - 1.344)	0.775	1.061 (0.812 - 1.387)	0.664

^1^Genotypes treated as categorical variables and compared to the reference category.

Model 1: unadjusted.

Model 2: adjusted for age, and gender.

Model 3: in addition adjusted for type 2 diabetes, body mass index, smoking, hypertension, LDL-C, HDL-C, TG

**Figure 2:**
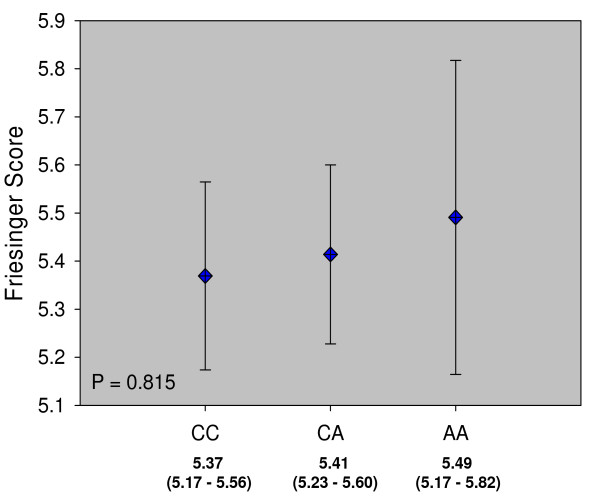
**Friesinger score according to rs2259816 genotype**. Friesinger score by rs2259816 genotype. Displayed are the estimated marginal means (± 95% confidence intervals) of a linear model (ANOVA) adjusted for age, gender, smoking (never, former, current), body mass index, diabetes mellitus, hypertension, LDL-C, HDL-C and TG.

We further looked for an association of rs2259816 with the friesinger score, a quantitative measure of the severity of CAD. ANOVA analysis didn't show a significant difference between the three genotype groups (Figure 2).

## Discussion

Recently, the discovery that the reduction of CRP levels in individuals who are not considered to be at high risk for cardiovascular disease by National Cholesterol Education Program guidelines significantly decreased the rate of first major cardiovascular events as well as total mortality has renewed the interest in CRP as possible biomarker for deciding whether an individual should receive treatment [45,46]. The JUPITER trial showed the benefit of statin treatment even for individuals with high CRP but normal LDL-C [45,47].

As a large part of the variability of plasma CRP levels is due to genetic causes attempts have been made to identify the responsible genes.

Besides polymorphisms in the CRP gene itself other genes have been identified, one of them being the gene for transcription factor hepatocyte nuclear factor (HNF)-1α. Several polymorphisms in this gene have been linked to lower CRP [33,34]. We analyzed one of these polymorphisms, rs2259816, in the LURIC cohort. In LURIC CRP is increased in CAD patients compared to healthy controls but the increase is only significant for patients with acute coronary syndromes, not for patients with stable CAD [48]. For the rs2259816 polymorphism we found a highly significant association with circulating levels of CRP confirming other reports. Individuals homozygous for the rare A allele showed an approximately 25% lower CRP compared to individuals homozygous for the C allele. However, this SNP had no impact on the risk of CAD in LURIC. The odds ratio even increased marginally for heterozygous or homozygous carriers of the A allele compared to CC homozygotes but this increase did not reach statistical significance. On the other hand our study has only a limited power to detect small effects on the risk of CAD. The estimated marginal mean of CRP in CC homozygotes was 3.88 mg/L compared to 2.95 mg/L in AA homozygotes. According to König et al. [49], who described a log-linear relationship between CRP and CAD, this difference should translate into an OR for CAD of only 1.13. To increase our power we looked for an association of rs2259816 with the friesinger index which is a quantitative score of the severity of CAD. Although there is also a slight increase in friesinger score for carriers of the A allele this difference was not significant, strengthening the notion that there is no causal relationship between CRP and CAD.

These findings are in line with Grammer et al. who didn't find an association of polymorphisms in the CRP gene itself and CAD in LURIC [12] and support the view that CRP does not play a causal role in the development of CAD.

Nevertheless, we think that HNF-1α might play an important role as a link between metabolic and inflammatory pathways. As such it could be involved in the pathogenesis of atherosclerosis and further work is required to define the role of HNF-1α more exactly.

## Conclusions

In the LURIC Study cohort the A-allele of the rs2259816 polymorphism in the HNF1A gene is associated with decreased CRP but not with coronary artery disease. These results support the view that CRP does not play an active role in the development of CAD.

## Competing interests

The authors declare that they have no competing interests.

## Authors' contributions

MEK performed the statistical analysis and drafted the manuscript. TBG assisted in the statistical analysis and helped to draft the manuscript. WR performed the genotyping. MW conceived of the study and participated in its design and coordination and helped to draft the manuscript. All authors read and approved the final manuscript.

## Pre-publication history

The pre-publication history for this paper can be accessed here:

http://www.biomedcentral.com/1471-2350/11/157/prepub
